# Assessing Impact of Sensors and Feature Selection in Smart-Insole-Based Human Activity Recognition

**DOI:** 10.3390/mps5030045

**Published:** 2022-05-31

**Authors:** Luigi D’Arco, Haiying Wang, Huiru Zheng

**Affiliations:** School of Computing, Ulster University, York Street, Belfast BT15 1ED, UK; darco-l@ulster.ac.uk (L.D.); hy.wang@ulster.ac.uk (H.W.)

**Keywords:** activity recognition, smart insole, machine learning, window size optimisation, feature selection

## Abstract

Human Activity Recognition (HAR) is increasingly used in a variety of applications, including health care, fitness tracking, and rehabilitation. To reduce the impact on the user’s daily activities, wearable technologies have been advanced throughout the years. In this study, an improved smart insole-based HAR system is proposed. The impact of data segmentation, sensors used, and feature selection on HAR was fully investigated. The Support Vector Machine (SVM), a supervised learning algorithm, has been used to recognise six ambulation activities: downstairs, sit to stand, sitting, standing, upstairs, and walking. Considering the impact that data segmentation can have on the classification, the sliding window size was optimised, identifying the length of 10 s with 50% of overlap as the best performing. The inertial sensors and pressure sensors embedded into the smart insoles have been assessed to determine the importance that each one has in the classification. A feature selection technique has been applied to reduce the number of features from 272 to 227 to improve the robustness of the proposed system and to investigate the importance of features in the dataset. According to the findings, the inertial sensors are reliable for the recognition of dynamic activities, while pressure sensors are reliable for stationary activities; however, the highest accuracy (94.66%) was achieved by combining both types of sensors.

## 1. Introduction

Pervasive computing is changing how we interact with technology, to the point that the goal nowadays is to create fully automated systems that can assist users without the need for interaction. Human Activity Recognition (HAR) is the application that is having the most success in terms of modelling human behaviour. HAR can be defined as the method to analyse data from sensors and reconstruct the activity carried out by users based on the context in which they are placed [1]. The application of wearable sensors for HAR cover a wide area, including health monitoring [2], rehabilitation [3], fall detection [4], sport monitoring [5], and industrial applications [6]. Despite the success of HAR in multiple scenarios, the interest of the research community has been focused on the recognition of Activities of Daily Living (ADLs) [7,8], which are all those activities performed on a daily basis, ranging from functional mobility (often referred to as ambulation) to dressing, feeding, and maintaining personal hygiene. Multiple solutions, including image, environmental, and wearable sensors, can be integrated into HAR systems; however, the evolution of Micro-Electro-Mechanical Systems (MEMS) has favoured the diffusion of wearable devices, which are cost-effective and require slight space to deploy, and seamlessly integrate with the user’s daily life [9]. Identifying the sensors that can be used to create a wearable device is not a simple task, as it is necessary to take into account both the minimum requirements to recognise the activities but also the space it can require for the user. Generally, inertial sensors are more used, such as accelerometers and gyroscopes, as they allow for monitoring the speed change in spatial and angular terms; however, other solutions such as the global positioning system (GPS) [10] and pressure sensors [11] can be found in the literature. Considering as the final aim the improvement of the performance of a HAR system, the integration of many devices must be accepted, as they enhance the results using multi-modal information [12]; however, they affect the comfort of the user when wearing them [13]. Considering a real-life application, it must be taken into account that the user could regret or forget to wear the designed equipment, making the system useless, such as elderly people who are prone to forgetting things.

In this study, the use of smart insoles as wearable devices for HAR has been investigated, which combine various sensors, such as inertial sensors and pressure sensors, allowing the analysis of activity patterns while minimising the discomfort for the user. They do not require user intervention after installation, except to recharge them if they have batteries [14]. For the classification of the activities, the Support Vector Machine (SVM) machine learning algorithm was used, which was identified in several studies as the one with the best results in HAR [15,16,17]. In general, HAR algorithms are machine learning algorithms that require data to be structured before they can be processed. The data segmentation and feature extraction procedures are two often used preprocessing methods. Most state-of-the-art HAR systems, regardless of the sensors and algorithms utilised, have to cope with classification errors caused by the width of the sliding window [18]. For this purpose, in this study, a grid search approach to determine the optimal window size was applied to reduce potential classification errors introduced by the window size. In addition, an in-depth analysis of pressure and inertial sensors was conducted to determine the importance of these sensors in a smart insole-based HAR algorithm. Determining in which activity these sensors may have the greatest impact is important to determine which sensors need to be integrated when building a new system to improve performance. This investigation was conducted using a one-vs-all strategy focusing on activity-by-activity classification performance. A feature selection technique to identify the optimal subset of features to improve the classification was adopted.

The main contribution of this paper is to propose an improved smart insole-based HAR system by addressing the following aspects:

- Optimisation of data segmentation window size in HAR for pressure and inertial sensors-based smart insoles;
- Assessing the impact of inertial and pressure sensors in recognition of ambulation activities;
- Investigation through feature selection of an optimal subset of features and assessment of the most important sensors in smart insole-based HAR.

The rest of this paper is structured as follows. Related works are described in Section 2. The proposed methodology is presented in Section 3, followed by the findings discussed in Section 4. The paper is concluded by a summary in Section 5.

## 2. Related Work

Looking at the current scientific landscape of activity recognition, many works can be identified that incorporate all of the sensors required to execute this operation within the smart insoles.

Sazonov et al. [15] presented a shoe-based wearable sensors system combined with a mobile phone for real-time recognition of various postures/physical activities and the resulting energy expenditure. They involved five force-sensing resistors and a 3-axis accelerometer. The data were collected at 25 Hz (400 Hz, averaging 16 consecutive samples). The activities defined were: sitting, standing, walking/jogging, and cycling; which were classified by the means of three machine learning models: Support Vector Machine (SVM), Multinomial Logistic Discrimination (MLD), and Multi-Layer Perceptron (MLP). Instead of processing raw data, the mean, the entropy and the standard deviation were extracted from the collected data. The MLP and MLD reached almost the same performance as the SVM, which had an accuracy of about 96%, but reducing the execution time and the memory requirements by a factor of >$10^{3}$.

Dehzangi et al. [19] proposed a methodology for investigating the discriminative capability of 13 pressure sensors, as well as the fusion of several pressure sensors for improved activity monitoring. The data were collected at a sampling frequency of 50 Hz. To allow processing, the following features were extracted: STFT, Katz, AR, Max Values, Variance, Power, and Mean. The algorithms involved, K-Nearest Neighbour (KNN) and Support Vector Machine (SVM), were trained for the recognition of six different activities: Sitting, standing, walking, running, jumping, and cycling. The SVM was the optimal one with an accuracy of 91.8%; however, with the Multi-Layer Neural Network (MLNN) used to incorporate sensor fusion, the average accuracy reaches 97.63%.

McCalmont et al. [20] developed a framework for analysing and assessing human gait using smart insoles. The smart insole was composed of eight pressure sensors and nine degrees IMU sensors (accelerometer, gyroscope, and magnetometer). Raw data were processed and different features were extracted to allow the processing: mean, standard deviation of acceleration, velocity and total acceleration, and cadence. Five different activities were investigated: Slow walking, normal walking, fast walking, climbing upstairs, and walking downstairs. Three machine learning models were included to classify those activities: Artificial Neural Network (ANN), K-Nearest Neighbour (KNN), and Random Forest (RF). The best performance was reached by the ANN, which reached an accuracy of 80%.

Jeong et al. [17] exhibited a smart shoes-based ambulatory classification system. The smart shoes involved eight pressure sensors, which, using a Support Vector Machine (SVM), were exploited to classify three ambulation activities: level walking, stair descent, and ascent. The feature extracted from raw data were calculated according to the pressure intensity as the product between the pressure and the sampling time for each step epoch. Three participants were involved in the data collection, in which the sampling frequency was set to 50 Hz and window size set according to the number of steps, between one and six. The overall accuracy achieved by the proposed system was 95.2%.

All the studies reviewed yielded high results; however, none examined how the selection of a sliding window size could affect the final classification. Furthermore, these studies reveal how different sensors, such as pressure and inertial sensors, can be used and incorporated inside smart insoles, but no research has been conducted on the importance of the latter, and why they are required for optimal results.

## 3. Materials and Methods

Human activities can be recognised according to certain patterns that are repeated throughout time. In this research, the responsibility of recognising the activity patterns is delegated to a machine learning model, which autonomously establishes the recognition bounds. To lower the impact of such a system on the user’s daily life, a pair of smart insoles has been involved, with the aim of proving their effectiveness in HAR. Furthermore, many investigations have been made into the system’s optimisation and the evaluation of the sensors employed.

### 3.1. Sensing Elements

**Figure 1:**
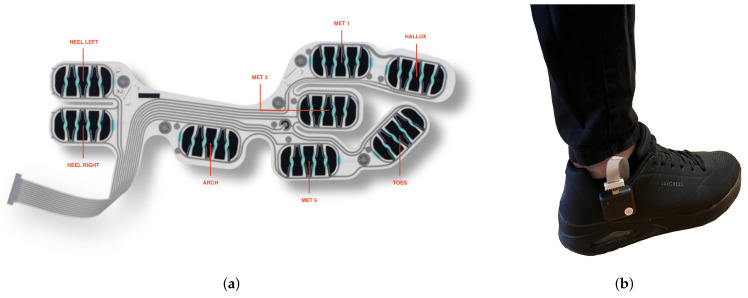
ActiSense Kit, IEE Luxembourg S.A. (**a**) IEE Smart Foot Sensor (**b**) Example of how the system is worn by the user.

To provide a minimally invasive HAR system for the user, a pair of smart insoles, from IEE Luxembourg S.A., were used in this study as the only device. The smart insole system is composed of two smart insoles and two electronic control units (ECU), as shown in Figure 1. The smart insole is made up of eight pressure cells that are situated where the impact foot-to-ground is higher (heel left, heel right, arch, met 1, met 3 met 5, hallux, and toes). The ECU incorporate a tri-axis accelerometer (range: ±8 G), tri-axis gyroscope (range: ±1000 DPS) and a tri-axis magnetometer (range: ±4912 μT), providing inertial measurement with nine degrees of freedom. The data acquired by the smart insoles are saved locally on a flash memory inside the ECU and are shared with a smartphone using the built-in Bluetooth Low Energy (BLE).

### 3.2. Data Collection

In this study, data were collected from five volunteers. The participants, aged from 25 to 55, reported no histories of lower limb injuries. Each participant was provided with one pair of the IEE smart insole kit (Figure 1a), which was inserted into their own common shoes, with the ECU firmly clicked on the sides of the shoes, as shown in Figure 1b. The data collection was conducted through an Android application via a BLE connection between the smartphone and the smart insole system. Participants wore the smart insoles on both feet, providing dual support information. The data from both feet were synchronised using the timestamp. The activities included in this study are six ambulation activities: ascending stairs, descending stairs, sitting to standing, sitting, standing, and walking on a treadmill (slow, normal, and fast, with a speed of 3.6, 4.7, 5.8 km/h, respectively). The sampling frequency for the data collection was set to 200 Hz and each participant carried out different activities in different environments due to the COVID-19 pandemic, and not all the participants completed the entire set. A total of about 120 minutes of data were recorded.

### 3.3. Data Segmentation

The data gathered with the help of the participants contained information regarding various activities, but each recording was different in size from the others, so the first step in processing the data was to define a dimension that could encompass every single activity in the dataset and allow data segmentation. Considering that the sensors’ data were in form of discretised, continuous data flow, the data segmentation can be generalised as follows: given a set $S=\{S_{0},...,S_{k-1}\}$ of *k* time series defined within a time interval $I=[t_{\alpha },t_{\omega }]$, the objective is to find a set of labels representing the activity performed in an a temporal partition $<I_{0},...,I_{r-1}>$ of *I*, such that the times intervals $I_{j}$ are consecutive, non-empty, non-overlapping, and respect ${⋃}_{j=0}^{r-1}I_{j}=I$ [21]. Concerning ambulation activities, which involve only the lower body part, it is reasonable to assume that the activities are not simultaneous and that each interval represents only one activity. The intervals $I_{j}$ can also be called *windows*; therefore, the first step in developing a classifier is to set the size of the window in order to contain one activity within it.

In literature, multiple approaches are reported for data segmentation, including Explicit windowing, Time windowing, Sensor Event windowing, and Dynamic windowing [22]. In this study, we focused on the application of Time windowing, because it allows the dataset to be split into multiple equal periods, adapting perfectly to the continuous data present in the dataset and preparing the system for future real-time applications. The challenge with this approach is determining the appropriate window size, because a window size that is too small will not accurately represent all of the activities involved, and a window size that is too large will include multiple activities in one window, impacting the final classification. Typically, empirical choices are made in the literature to set the window size based on the researcher’s experience or utilising existing state-of-the-art studies as a reference, for example, Merry et al. [16] proposed a window size of 0.5 s, Truong et al. [23] a window size of 10 s, and Ghosh et al. [24] a window size of 60 s.

In this study, a grid search strategy was used to discover the optimal window size. The HAR models were built using a machine learning algorithm on multiple datasets with different window sizes. This test employed windows ranging from 1 to 20 s in length, with one second off. Using a Stratified 5-fold cross-validation, the tests were repeated, and the window size with the greatest score and the least deviation in results was determined to be optimal.

### 3.4. Feature Extraction

After data segmentation, the raw data were transformed into a set of feature vectors using feature extraction. Each instance in the processed dataset corresponded to the feature vector extracted from all the signals within a time window. In this study, two types of features were extracted according to the data domain: time-domain features and frequency-domain features. The time-domain features allowed analysing the signals in reference to time, and instead, the frequency-domain features allowed analysing the signals in reference to frequency.

Table 1 presents the features used in this study. It shows eight features that were extracted from each time series generated by the used sensors, five from the time domain and three from the frequency domain. The smart insole has eight pressure sensors, a 3-axis accelerometer, a 3-axis gyroscope, and a 3-axis magnetometer thus creating 17 different time series per insole. The final number of features extracted from the dataset was: 17×8×2=272 where 17 is the number of time series per insole, 8 is the number of features and 2 is the number of insoles involved.

### 3.5. Activity Recognition Algorithm

In this study, the Support Vector Machine (SVM) machine learning algorithm was employed for HAR. It is a supervised learning method that generates an input–output mapping function from a set of labelled training data [30]. It is a kernel-based algorithm that can be adapted for linear to non-linear cases. There are many kernel functions such as the linear, the polynomial, the radial basis function or the gaussian kernel and the sigmoid. The kernel chosen in this paper was linear, as it has been proven to be the best performing one in experiments.

The SVM algorithm has also been applied widely in previous studies [15,16,17], demonstrating that it can provide excellent results and the ability to outperform other machine learning techniques, thanks to the ability to create hyperplanes, which allow for obtaining a correct separation of the data relating to each activity.

All findings reported in this manuscript refer to the outcomes of experiments using the SVM. Each experiment was carried out using the Stratified 5-fold cross-validation [31], which divides the data into five parts while preserving the balance of the samples belonging to the respective classes in the original dataset; then, one part is reserved for testing and all the others are used for training. Due to the number of data samples in the minority classes, 5-fold cross-validation was selected to ensure a sufficient representation of a specific minority class in both training and test sets. Furthermore, we have experimented with different numbers of folds for the validation, and there was no significant difference between using 5 folds and a higher one such as 10 folds.

### 3.6. Assessment of Impact of Sensors

The smart insoles used in this study consist of two types of sensors, including pressure sensors and inertial sensors. Understanding the impact that each type of sensor has in the HAR can define the role and the importance of that specific sensor in the classification. This evaluation can further be exploited to reduce the amount and types of sensors involved in a new system when a different subset of activities is defined.

The assessment has been carried out by defining three different datasets created using a different combination of sensors: only pressure sensors, only inertial sensors, and the combination of pressure and inertial sensors. The SVM was trained on all the datasets using a one-vs-all strategy, using the Receiver Operating Characteristic Curve (ROC-Curve) as metric for evaluating how well the model can differentiate one class against the others. The ROC-Curve is made by plotting the True Positive Rate (TPR) on the y-axis against the False Positive Rate (FPR), which can be computed as follows:

(1) $$\mathrm{TPR}=\frac{\mathrm{TP}}{\mathrm{TP}+\mathrm{FN}}$$

(2) $$\mathrm{FPR}=\frac{\mathrm{FP}}{\mathrm{FP}+\mathrm{TN}}$$

where TP represents the True Positive, FP the False Positive, TN the True Negative, and FN the False Negative.

The Area Under the Curve (AUC), which measures the two-dimensional area beneath the entire ROC curve, was used in conjunction with the latter to summarise the ROC-Curve in order to compare the various experiments, as well as the recognition of the activities between them.

### 3.7. Feature Selection

Machine learning methods have difficulty in dealing with a large number of input features. Hence, a process is needed to remove unneeded features from the dataset. This process is called Feature Selection and allows for identifying relevant features, or a candidate subset of features aiming at the reduction of unnecessary, redundant, or noisy data. Feature selection can speed up a data mining algorithm, improving learning accuracy, and leading to better model comprehensibility. Irrelevant features provide no valuable information, while redundant features supply no further information to the currently selected features [32]. Since the dataset used in this research has 272 features, using the feature selection procedure to reduce the number of features is one way to improve the classifier’s performance and/or reduce the computational time. The type of feature selection algorithm used in this study is the Univariate Selection [33], which is a type of feature selection that bases its operation on the calculation of the statistical relationship between the input variables and the output variables. The ANOVA (Analysis of Variance) [34] scores are used for the evaluation of the features’ scores, as it is a set of statistical techniques that allows for comparing two or more groups of data by comparing the variability within these groups with the variability between groups. Defining a number *k*, which is the desired number of features to be selected, the ANOVA method can determine the best subset of *k* highest score elements. Hence, a grid search strategy was applied to define the best value for k. The 272 subsets were created using a number of features ranging from 1 to 272. After training the 272 SVM on each subset and comparing the performance (F1-Score), the optimal subset with 227 features was identified as the one with the best performance and the fewest features.

The feature selection, in this study, was used not only for improving the classification but also to determine the importance of each sensor by assessing the total number of features selected for each sensor.

## 4. Results and Discussion

This study proposed a smart insole-based HAR system for daily life usage. To optimise and explain the results achieved, multiple techniques have been applied, including optimisation of the data segmentation window size, analysis of sensors and features importance, and feature selection.

### 4.1. Data Segmentation Optimisation

**Figure 2:**
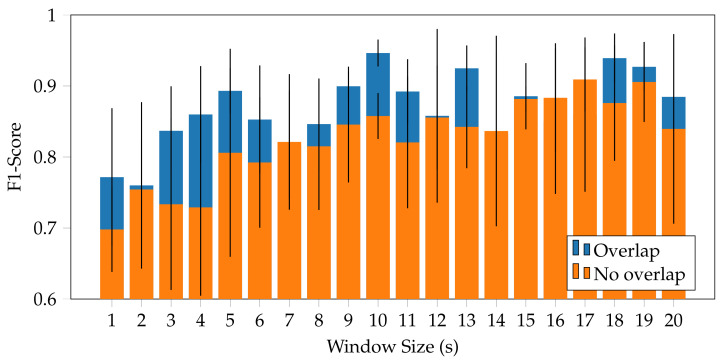
Result of the grid search strategy to identify the optimal sliding window size for classification. The 10 s window size with 50% of overlap achieved the highest *F1-Score* (94.64%). Overall in almost all the window sizes tested, the introduction of the overlapping between consecutive windows allowed an increase in the classification performance.

The optimisation of the window size for data segmentation followed a grid search approach to identify among a series of predefined window sizes (ranging from 1 to 20 s with one second off) the one that could improve the performance of the classifier. From the evaluation, the best window size was the 17 s time window with an *Accuracy* of 91.39% and an *F1-Score* of 90.92%. However, introducing the 50% oversampling technique, to reduce the information loss at the edges of the window, the optimal window resulting was 10 s with an *Accuracy* of 94.66% and an *F1-Score* of 94.64%, as shown in Figure 2. This time window is able to take into account the cadence of an activity, such as the pace of the walking activity, considering an average of 5 steps. Furthermore, the introduction of the overlap between consecutive windows allows the recognition of the activities that could occur between two different windows. Figure 2 shows the performance obtained with each window size under examination, showing how the use of overlap allows in most cases one to increase the performance of the model proposed.

### 4.2. Assessment of Sensors’ Impact in HAR classification

Investigating the significance of the sensors for the activity recognition is required to refine the model and establish which sensor has the greatest impact on recognition. The pressure sensors, the inertial sensors, and the combination of both have been tested separately to assess the capability of each sensor in the classification of each activity.

**Figure 3:**
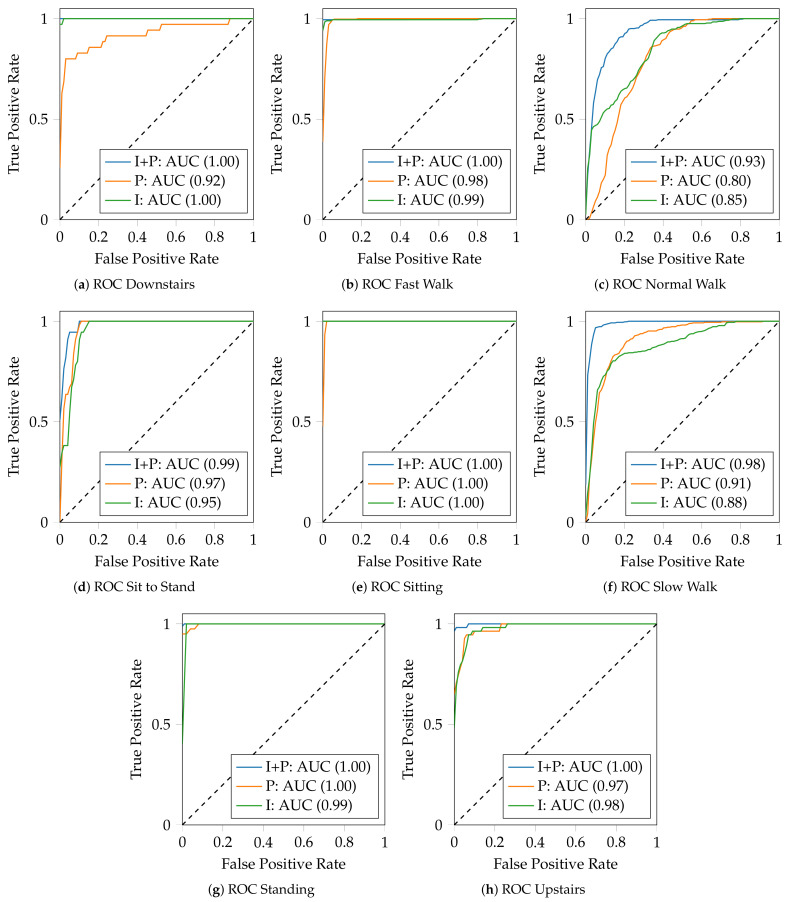
Evaluation of ROC curves for each activity using three different datasets built from different types of sensors: Inertial sensors and pressure sensors (I + P), pressure sensors (P), and inertial sensors (I). In (**e**), since the sitting activity is correctly classified in all the experiments, the curve “I+P: AUC (1.00)” overlaps “I: AUC (1.00)” and is not visible.

Figure 3 presents the findings obtained for each activity using each type of sensor, including both the *ROC-Curves* and the *AUC scores*.

According to the results, the only activity that has been correctly categorised in all of the investigations is sitting (Figure 3e). Inertial sensors performed better in the following activities: downstairs, fast walk, normal walk, and upstairs, with an *AUC score* of 100%, 99%, 85%, and 98%, respectively. Pressure sensors did better, instead, in the following activities: sit to stand, slow walk and standing, with an *AUC score* of 97%, 91%, and 100%, respectively. The combination of the inertial and pressure sensors, on the other hand, outperformed the other two experiments in all activities, demonstrating how it is fundamental to combine multi-modal information from multiple sensors for recognising human activities.

Inertial sensors provide high differentiate capabilities in the recognition of dynamic activities reaching high performance, as shown in the downstairs activity (Figure 3a), making them completely self-sufficient in this specific application. On the other hand, however, pressure sensors provide high performance for the recognition of stationary activities, where the distribution of the user’s weight on the foot affects the classification, maximising performance, but also in activities in which the rhythm in carrying out the activity is slow, such as slow walking (Figure 3f), in which the inertial sensors can be misled.

Considering systems in which the reduction of the costs is a primary requirement, both types of sensors can be used individually, depending on the type of activity to be recognised, to save costs on hardware and processing. However, if the set of activities involved in the study comprises both dynamic and stationary activities, the combination of both types of sensors, pressure and inertial sensors, is preferred, as it is more reliable and provides higher performance.

### 4.3. Selection and Assessment of Features

To optimise the performance of the proposed classifier, but at the same time determine the sensors with the greatest importance for classification, a feature selection technique was applied to eliminate those features that are not significant or redundant.

All possible subsets of features (ranging between 1 and 272) were evaluated using an approach that evaluates the statistical relationship between a subset of features and the expected output, through the use of an ANOVA score to determine the subset with the minimum number of features and the greatest performance. The findings demonstrated that the minimum number of features to be included in the final system was 227, which kept the classifier performance unchanged, achieving an *Accuracy* of 94.66% and an *F1-Score* of 94.64%. The advantages of this feature reduction are not only related to the classifier, but also to the preprocessing operations, which will have to manage a smaller amount of data and thus boost the execution speed, which is a prerequisite for future integration in a real-time system.

**Figure 4:**
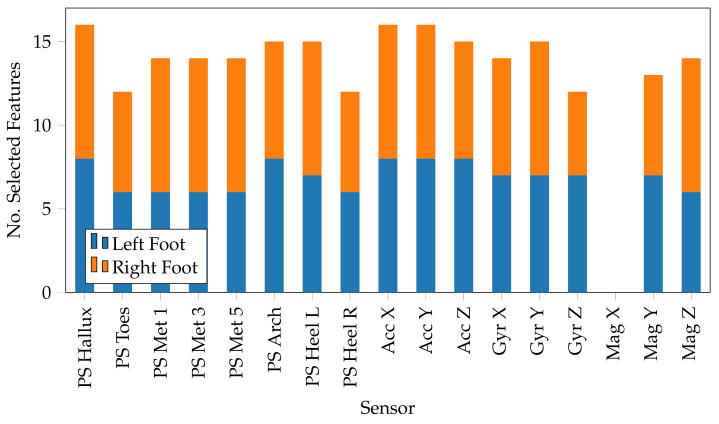
Number of features selected by each foot for each sensor. The hallux is the most relevant pressure sensor (*PS*), whereas, the accelerometer (*Acc*) is the most significant inertia sensor, followed by the gyroscope (*Gyr*). The x-axis of the magnetometer (*Mag*) is the only sensor in this investigation that had no bearing on the activity classification.

Furthermore, the major contribution of this feature selection can be traced back to the evaluation of the features chosen for each sensor, determining the value of each sensor in activity classification based on the number of features employed originating from that sensor. According to Figure 4, the hallux sensor was the most important among the pressure sensors, with a total of 16 features selected, followed by the arch and the heel left sensors. The heel sensor is generally recognised as the most important sensor [19], due to its key role within the gait cycle; however, the hallux sensor can be recognised with the same importance, as before a swing, the hallux is the last area of the foot to make contact with the ground, resulting in a high-pressure change. Furthermore, considering activities, such as ascending and descending stairs, the hallux sensor has a greater value, since some people tend not to support the heel in these activities and to put all their weight on the front of the foot. This is reflected in the results shown in Figure 4. The accelerometer stands out among the inertial sensors, particularly on axes x (roll angle) and y (pitch angle). The lack of activities that incorporate twisting movements, in which the yaw angle is critical for detection, can be linked back to this finding. Overall the features selected tend toward inertial sensors against pressure sensors, except for the x-axis of the magnetometer, which has no essential importance and is not included in the final set. In terms of the number of features selected per foot, the right side was chosen 116 times against 111 times for the left side. The right side has a slightly higher value, which may be due to the demography of the participants in this study, in which there is a high concentration of right-handed. Thus, participants may have placed more emphasis on the right side when performing the activities, thereby increasing the importance of the latter. However, a study to evaluate left-right dominance will be addressed in a further study.

### 4.4. Performance Evaluation and Limitations

The proposed system made it possible to demonstrate the validity of smart insoles for the recognition of activities, but also to evaluate the best configurations to optimise the latter according to the type of activities involved.

The proposed system achieved high performance, reaching an *Accuracy* and an *F1-Score* of 94.66% and 94.64%, respectively. By combining the pressure and inertial sensors, it was possible to build a system that fully exploits the presence of multi-modal information, which has proven to be able to achieve high performance, as reported in Table 2. The analysis of the types of sensors made it possible to determine the importance that each has in the classification, demonstrating the pressure sensors fundamental in the recognition of stationary activities, and inertial sensors in the recognition of dynamic activities. The use of a feature selection technique then made it possible to identify the importance of each individual sensor, especially for inertial sensors, in which the use of both accelerometer, gyroscope, and magnetometer is necessary.

Although the performances obtained are excellent, it is important to highlight the limitations of this study and how they will be addressed in the future. Data collection and analysis occur at different times, making integration inside a real-time application problematic at the moment. However, by addressing issues such as synchronisation and data storage, it will be feasible to integrate the system into such applications.

In this study, the data collection sampling frequency was set to 200 Hz; however, it may result in high power consumption for the device. Previous studies [35] demonstrated that a low sampling rate can save energy assumption while maintaining reasonable HAR performance when using IMU sensors. However, limited study has been undertaken on assessing the impact of sampling rates of smart insole-based HAR systems. This deserves further investigation in the future.

The dataset used for this study was created on the data collected by five volunteers, who collected the data in a completely independent way and personally labelled the activities carried out, thus creating noise within the dataset when an activity has not been completely isolated. Furthermore, the number of participants should be increased to include data from more heterogeneous people, for mining more patterns inside the data and clearly assessing whether different participant characteristics can affect the findings, such as the relationship between the size of the time window and the age of the participants. Hence, a future work will be undertaking data collection sessions that are supervised by an expert and increasing the participant number to have a more heterogeneous cohort. Due to the grid search approach, the best window size has been set to 10 s with a 50% overlap; nevertheless, an investigation into which activity has the major influence on this outcome will be conducted. The activities that occur between two consecutive windows have been managed using the sliding window with overlap, but a more in-depth study is necessary to be able to classify even the transitory activities, such as the transition between standing and sitting.

## 5. Conclusions

In this study, a smart insole-based HAR system was presented, investigating the impact of sensors, time window size, and features on the activity recognition using SVM. The smart insole, which consists of pressure sensors and inertial sensors, has been proven to be effective in recognising ambulation activities such as downstairs, sitting, sit to stand, standing, upstairs, and walking, with an overall *Accuracy* and *F1-Score* of 94.66% and 94.64%, respectively. The classification was handled by a machine learning model, the Support Vector Machine (SVM), which used features extracted from both the time and frequency domains. The study focused on optimising the data segmentation window size to avoid classification errors caused by the improper temporal window size. The 10 s with 50% overlap was found to be the best option, which used a grid search approach. An investigation of the types of sensors incorporated into the smart insole for HAR uncovered the importance of pressure and inertial sensors in reducing misclassification errors, making them necessary for high performance. However, pressure sensors individually demonstrated good performance in recognising stationary activities, with inertial sensors in dynamic activities. To improve the performance of the system, a feature selection technique was applied to the dataset, detecting the optimal subset to be comprised of 227 features. The findings revealed a slight predominance of the right foot over the left in terms of the overall number of selected features, but further study is required to generalise this concept, as the data was collected mainly from right-handed people. Furthermore, the hallux turned out to be the most important pressure sensor, while the accelerometer is the most important inertial sensor. Although the study is carried out using the commercially available smart insole kit from IEE Luxembourg S.A, the HAR algorithm proposed in this study can be applied to other smart insole devices. Considering the integration and the benefits that an activity recognition system based on smart insoles can bring in a scenario of daily life, a future study will focus on the transformation of this system for the collection and processing of data in real-time, dealing with problems such as data synchronisation and data storage.

## Figures and Tables

**Table 1:** Summary of features extracted from data series generated by each sensor (*x*) per each window of size *N*. The frequency-domain features were calculated using the Fourier Transform coefficients ($F_{i}$).

Domain	No.	Name	Equation	Description	References
Time	1	Mean	$\mu \left(x\right)=\frac{1}{N}\sum_{i=1}^{N}x_{i}$	Average of the data	[15,19,20,25,26,27,28,29]
	2	Range	r(x)=max(x)−min(x)	Difference between the greatest and the smallest values in the data	[29]
	3	Standard Deviation	$\sigma \left(x\right)=\sqrt{\frac{1}{N}\sum_{i=1}^{N}{\left(x_{i}-\mu \right)}^{2}}$	Measure of dispersion in the data	[15,20,25,27,29]
	4	Skewness	$s\left(x\right)=\frac{1}{N\sigma^{3}}\sum_{i=1}^{N}{\left(x_{i}-\mu \right)}^{3}$	Measure of asymmetry of a distribution around its mean	[25,26,28]
	5	Kurtosis	$k\left(x\right)=\frac{1}{N\sigma^{4}}\sum_{i=1}^{N}{\left(x_{i}-\mu \right)}^{4}$	Measure of how different a distribution’s tails are from the tails of a normal distribution	[25,26,28]
Frequency	6	DFR	$DFR\left(x\right)=\frac{max(\left\{F_{1},...,F_{n}\right\})}{\sum_{i=1}^{N/2}F_{i}}$	Dominant Frequency Ratio, ratio of highest magnitude FFT coefficient to the sum of magnitudes of all FFT coefficients	[29]
	7	Entropy	$entropy\left(x\right)=-\sum_{i=1}^{N/2}F_{i}\log_{2}F_{i}$	Information entropy of the normalised values of FFT coefficient magnitude	[15,29]
	8	Energy	$energy\left(x\right)=\sum_{i=1}^{N/2}F_{i}^{2}$	Sum of the squared discrete FFT component magnitudes	[19,25,28]

**Table 2:** Summary of classification results of using the SVM and a stratified 5-fold cross-validation on different datasets. Values in the columns are the average value and the standard deviation (in parenthesis) of the corresponding performance metric.

Sensors	Accuracy	Precision	Sensitivity	F1-Score
**IMU + Pressure**	94.66 (±2.08)	95.09 (±1.91)	94.66 (±2.08)	94.64 (±2.12)
IMU	72.70 (±19.63)	79.50 (±15.17)	72.70 (±19.63)	68.86 (±24.17)
Pressure	85.26 (±5.69)	88.29 (±3.82)	85.26 (±5.69)	84.77 (±6.37)

## Data Availability

Not applicable.
